# Essential and Non-Essential Amino Acids in Dogs at Different Stages of Chronic Kidney Disease

**DOI:** 10.3390/vetsci9070331

**Published:** 2022-06-30

**Authors:** Ilaria Lippi, Francesca Perondi, Alessio Pierini, Francesco Bartoli, Eleonora Gori, Chiara Mariti, Veronica Marchetti

**Affiliations:** 1 Dipartimento di Scienze Veterinarie, Università di Pisa, 56122 Pisa, Italy; ilaria.lippi@unipi.it (I.L.); f.perondi87@gmail.com (F.P.); pierini.alessio2004@gmail.com (A.P.); chiara.mariti@unipi.it (C.M.); veronica.marchetti@unipi.it (V.M.); 2 Dipartimento di Ricerca Traslazionale e delle Nuove Tecnologie in Medicina e Chirurgia, 56120 Pisa, Italy; francesco.bartoli@med.unipi.it

**Keywords:** essential amino acids, non-essential amino acids, CKD, metabolic acidosis, CaxP product, PEW

## Abstract

**Simple Summary:**

Human patients affected by chronic kidney disease reported alterations of serum amino acid pattern, as a consequence of several metabolic complications of the renal disease. Our study aimed to evaluate possible differences in serum pattern of essential and non-essential amino acids between dogs with chronic kidney disease and clinically healthy subjects. Canine chronic kidney disease resulted in an association with a significant serum deficiency of the majority of both essential and non-essential amino acids. The amino-acidic disorder was particularly evident when protein-energy wasting syndrome was present, suggesting a potential key role of pathological conditions, such as inflammation, insulin resistance, and increased protein breakdown.

**Abstract:**

Abnormalities of serum amino acid profile, mostly characterized by a reduction in essential amino acids (EAAs) and an increase in non-essential amino acids (NEAAs), have been documented in human chronic kidney diseases (CKD). Amino acid disorders have been associated with CKD complications, such as metabolic acidosis and malnutrition. The aim of the present study was to evaluate EAAs and NEAAs in dogs affected by CKD at different IRIS stages, with particular reference to calcium–phosphate abnormalities, metabolic acidosis, and protein-energy wasting syndrome (PEW). Serum EAAs (L-histidine, L-isoleucine, L-leucine, L-lysine, methionine, L-phenylalanine, L-threonine, tryptophan, L-valine, and L-arginine) and serum NEAAs (L-alanine, L-aspartic acid, L-cysteine, L-glutamic acid, glycine, proline, L-serine, and L-tyrosine) were analyzed with HPLC in a group of dogs with CKD (*n* = 62), and in a group of healthy dogs (*n* = 25). CKD dogs showed significantly lower serum levels of histidine (*p* < 0.000), isoleucine (*p* < 0.000), tryptophan (*p* < 0.000), alanine (*p* = 0.013), cysteine (*p* < 0.000), and serine (*p* = 0.002), and significantly higher levels of proline (*p* < 0.000), leucine (*p* = 0.001), lysine (*p* < 0.000), valine (*p* < 0.000), arginine (*p* = 0.002), glutamic acid (*p* = 0.002), and glycine (*p* = 0.010) compared to healthy dogs. Dogs with abnormal calcium x phosphate values showed significantly higher levels of cysteine (*p* = 0.003), and lower levels of tryptophan (*p* = 0.025) compared to CKD dogs with normal CaxP. Dogs with metabolic acidosis showed significantly higher levels of phenylalanine (*p* = 0.035) and leucine (*p* = 0.034) compared to CKD dogs without metabolic acidosis. Dogs with PEW showed significantly lower levels for most of amino acids. In PEW dogs, the median distribution of both EAAs (*p* = 0.000) and NEAAs (*p* = 0.001) was significantly lower. The serum pattern of both EAAs and NEAAs was significantly different in CKD dogs compared to healthy dogs, although no association with the progression of the IRIS stage was found.

## 1. Introduction

In human chronic kidney disease (CKD) patients, abnormalities of serum amino acid profile have been extensively documented, with a reduction in essential amino acids (EAAs) and an increase in non-essential amino acids (NEAAs) concentrations [1]. Data regarding EAA and NEAA serum pattern in CKD dogs are scarce, and mostly limited to later stages of the disease, or to particular conditions [2,3,4,5]. Serum amino acid profile has been investigated in dogs with protein losing nephropathy, and in cats with CKD [3,4]. These dogs experienced significant reduction in some EAAs (leucine, threonine, and histidine) and some NEAAs (glycine, proline, asparaginase, tyrosine, hydroxyproline, and serine) compared to control dogs. Although serum reduction in leucine, tyrosine, and threonine was a common finding in both dogs with protein losing nephropathy and CKD humans, several dissimilarities between the two species were found. CKD people showed constantly elevated serum concentrations of NEAAs [3].

Several abnormalities occurring during CKD, such as uremia, metabolic acidosis, and malnutrition, may contribute to disorders in serum and muscle amino acid profiles [1]. Patients with inadequate serum concentrations of EAAs may be unable to meet physiologic demands for protein synthesis, thus increasing the risk for hypoalbuminemia, loss of lean body mass, and protein malnutrition [2]. Cachexia has been historically associated with human CKD, with an estimated prevalence of 20%. Although the exact prevalence of cachexia in CKD dogs is unknown, it is considered a common complication, with significant negative effects on progression of the disease and survival. In renal failure, cachexia results from a condition of negative energy intake, and increased protein catabolism, as a consequence of the concomitant presence of metabolic acidosis, inflammation, and insulin resistance. Beside these disorders, abnormalities in the intestinal absorption of proteins, dysbiosis, may contribute to cause derangements in serum amino acid concentrations [5,6].

The aim of the present study was to evaluate EAA and NEAA serum profile in dogs affected by CKD at different IRIS stages, with particular reference to calcium–phosphate (CaxP) abnormalities, metabolic acidosis, and protein-energy wasting syndrome (PEW).

## 2. Materials and Methods

Medical records showing a diagnosis of CKD were reviewed for client-owned dogs of different breed, sex, and weight, and referred to the nephrology and urology service of the Veterinary Teaching Hospital “Mario Modenato” of the University of Pisa, between January 2020 and January 2022,. Dogs were considered eligible for the study if they met at least one of the following criteria: (1) azotemic dogs (serum creatinine > 1.5 mg/dL) with documented CKD (based on historical, clinical, ultrasonographical, and biochemical findings); (2) non-azotemic dogs with PU/PD, serum symmetric dimethyl arginine (SDMA) >18 μg/dL, and ultrasonographical signs of CKD (irregular kidney shape, reduced cortico-medullary distinction, and hyperechoic cortices). Included dogs were classified according to the IRIS guidelines. According to serum creatinine and SDMA values, CKD dogs were divided in four groups: IRIS stage 1, IRIS stage 2, IRIS stage 3, and IRIS stage 4. Dogs with historical, clinical, biochemical, and ultrasonographical signs of acute kidney injury (AKI) were excluded from the study. For each dog, data regarding history, physical examination, biochemical profile, urinalysis, and abdominal ultrasonography were collected from the medical record. For the CKD group, exceeding serum samples were stored (−80 °C) with the owner’s informed consent and used to assess amino acids.

A group of clinically healthy dogs (*n* = 25), of various sexes, breeds, and ages, was included in the study, with the owners’ informed consent, between January 2020 and January 2022. Dogs of the control group were enrolled among blood donors and were all clinically healthy with normal blood work and no previous history of medical issues. Serum samples used for determination of amino acids derived from exceeding serum aliquots, which are routinely stored (−80 °C) for research purposes.

Among essential amino acids, the following were analyzed: L-Histidine (HIS), L-Isoleucine (ILE), L-Leucine (LEU), L-Lysine (LYS), Methionine (MET), L-Phenylalanine (PHE), L-Threonine (THR), Tryptophan (TRP), and L-Valine (VAL). Among non-essential amino acids, the following were analyzed: L-Alanine (ALA), L-Arginine (ARG), L-Aspartic acid (ASP), L-Cysteine (CYS), L-Glutamic acid (GLU), Glycine (GLY), Proline (PRO), L-Serine (SER), and L-Tyrosine (TYR). Amino acids were analyzed with an automated high-performance liquid chromatography (HPLC) amino acid analyzer (AAA AccQ-Tag Test^®^, Merck Millipore Corporation), using a previously described method [7].

CKD dogs were subclassified according to serum calcium–phosphate product (CaxP), presence of protein-energy wasting syndrome (PEW), and metabolic acidosis. According to CaxP values, dogs were classified into normal (CaxP ≤ 70 mg^2^/dL^2^) and abnormal (CaxP > 70 mg^2^/dL^2^) categories [8]. As no clear definition of PEW in CKD dogs is available, PEW was defined in agreement with human guidelines [9] as the concomitant presence of serum hypoalbuminemia (≤2.5 g/dL), reduction in body weight, and daily caloric intake < RER. Reduction in body weight was considered significant for a 5% weight loss over 3 months, or a 10% weight loss over 6 months. RER was calculated by the following formula: 70 × (Kg of body weight)^0.75^ [10]. Metabolic acidosis was defined as a condition of blood pH < 7.35, and bicarbonate < 18 mmol/L [11].

Continuous variables were tested for normality by the Kolmogorov–Smirnov normality test. Normally distributed variables were expressed as mean ± standard deviation, and non-normally distributed variables were expressed as median and range. Serum concentrations of each amino acid were compared among the study groups by one-way ANOVA, or Kruskal–Wallis and Bonferroni post-test, according to their distribution. Median distributions of the sum of EAAs and NEAAs were compared among the study groups by Kruskal–Wallis and Bonferroni post-test. In the CKD group, serum concentrations of each amino acid and the sum of EAAs and NEAAs were compared by *t*-test or Mann–Whitney, between dogs with normal and abnormal CaxP, between dogs with and without PEW, and between dogs with and without metabolic acidosis, according to their distribution. Statistical significance was set for *p* < 0.05.

## 3. Results

The CKD group was composed of 62 dogs, which were distributed in IRIS stage 1 (*n* = 12), IRIS stage 2 (*n* = 16), IRIS stage 3 (*n* = 14), and IRIS stage 4 (*n* = 20). CKD dogs were composed of 26 females (42%), and 36 males (58%). Mean body weight was 17.9 ± 8.4 kg, and mean age was 8.3 ± 3.1 years. Median BCS and MCS were, respectively, 4.5 (range 2–7) and 2.1 (range 1–3). According to breed, the majority of dogs were mix-breed (*n* = 38; 62%), followed by Italian Spinone (*n* = 3; 6%), Shar-Pei (*n* = 3; 6%), American Staffordshire Terrier (*n* = 3; 6%), Dachshund (*n* = 3; 6%), Beagle (*n* = 2; 3%), Maltese (*n* = 2; 3%), German Shepherd (*n* = 1; 1%), Miniature Schnauzer (*n* = 1; 1%), Bull Terrier (*n* = 1; 1%), Pointer (*n* = 1; 1%), Boxer (*n* = 1; 1%), Yorkshire Terrier (*n* = 1; 1%), Pomeranian (*n* = 1; 1%), and Springer Spaniel (*n* = 1; 1%).

Median serum values of creatinine, urea, calcium, phosphate, CaxP, total protein, albumin, bicarbonate, and pH of CKD dogs at different IRIS stages are reported in Table 1.

The control group (CG) was composed of 25 dogs, which comprised 14 females (54%), and 12 males (46%). Mean body weight was 25.5 ± 4.2 and mean age was 3.5 ± 1.5 years. According to breed, the majority of dogs (*n* = 10; 40%) were mix-breed, followed by Labrador Retriever (*n* = 3; 12%), Golden Retriever (*n* = 3; 12%), English Setter (*n* = 2; 8%), Maremma Shepherd (*n* = 2; 8%), Border Collie (*n* = 2; 8%), Bernese Mountain Dog (*n* = 2; 8%), and Lagotto Romagnolo (*n* = 1; 4%). Median BCS and MCS were, respectively, 6 (range 5–7), and 3. Results of the one-way ANOVA, and Kruskal–Wallis comparison of serum amino acid concentrations among CG and CKD dogs at different IRIS stages are reported in Table 2.

In CKD dogs, abnormal CaxP was found in 22 dogs, which were distributed in IRIS 1 (*n* = 2; 9.1%), IRIS 3 (*n* = 4), and IRIS 4 (*n* = 16). Metabolic acidosis was diagnosed in 30 dogs, which were distributed in IRIS 1 (*n* = 2), IRIS 2 (*n* = 5), IRIS 3 (*n* = 5), and IRIS 4 (*n* = 18). PEW was present in 11 dogs, which were distributed in IRIS 2 (*n* = 1), IRIS 3 (*n* = 3), and IRIS 4 (*n* = 7).

Results of the comparisons of serum amino acid concentrations are reported in Table 3: between dogs with normal/abnormal serum CaxP, between dogs with and without metabolic acidosis, and between dogs with and without PEW.

## 4. Discussion

In the present study, dogs with CKD showed abnormal status of several essential and non-essential serum amino acids compared to clinically healthy dogs. Previous studies in humans reported a typical reduction in EAAs and an increase in NEAAs in CKD [2]. In our study, not all EAAs and NEAAs showed the same pattern. The median distribution of EAAs and NEAAs was not significantly different among the study groups. This finding seemed to reflect what was found by Parker and colleagues in dogs with protein-losing nephropathy, in which the ratio between EAAs and NEAAs did not differ significantly between healthy dogs and the CKD dogs with protein-losing nephropathy [3]. In human CKD patients, the reduction in EAAs is mainly related to a reduction in food ingestion [5]. Our population of CKD patients also included dogs in early stages of the disease, which were able to cover their caloric needs entirely from a renal diet. Therefore, it is plausible that our CKD dogs received an adequate amount of EAAs from their diet.

Among essential amino acids, HIS, ILE, and TRP decreased significantly, although their concentration did not seem to be affected by the severity of the IRIS stage. The reduction in HIS might be caused by different pathological conditions associated with CKD. Lower serum levels of HIS were found in CKD human patients, mostly associated with inflammation, increased oxidative stress, and PEW [2]. HIS was seen to be implicated in the globin synthesis and erythropoiesis, and oral or intravenous administration of HIS showed positive effects on the correction of anemia [12]. In addition to its role as a co-stimulating factor of erythropoiesis, HIS has also been shown to be an efficient scavenger of ROS [13]. In particular, HIS supplementation seemed to increase the expression and activity of both catalases and glutathione peroxidase, thus enhancing the antioxidant response [12]. In our study, PEW might play a significant role in reducing serum HIS. Dogs of the PEW group showed significantly lower serum levels of HIS compared to the nPEW group. This finding was similar to previous reports in human medicine, in which serum HIS values were significantly lower in PEW. This syndrome has been frequently recognized in CKD patients, and it is considered one of the most important causes of amino acid deficiency [2]. Although in our study PEW dogs showed the lowest serum levels of HIS, mean HIS was significantly lower in the entire CKD population, compared to clinically healthy dogs. Therefore, it is plausible that causes other than PEW (such as inflammation and oxidative stress) were responsible for reduction in serum HIS levels. Similarly, serum levels of ILE and TRP were reduced significantly in CKD dogs, compared to healthy dogs. This was an expected finding, as both serum ILE and TRP were found to be lower in uremia [14]. In uremic patients, TRP depletion has been associated with an increase in TRP catabolites, and it has been strongly associated with the loss of residual renal function [15]. TRP depletion may also be a consequence of a condition of microbiome dysbiosis. In the course of CKD, the intestinal environment has been shown to progressively change from a symbiotic to a dysbiotic state [16]. Among TRP catabolites, a particular importance is given to indoxyl sulfate, which represents a well-known uremic toxin [17]. In CKD the reduction in TRP and ILE has also been associated with malnutrition [14]. The potential key role of malnutrition in the reduction in serum levels of TRP and ILE also seemed to be confirmed by our results, in which TRP and ILE were significantly lower in the PEW group.

In our cohort, not all the branched-chain amino acids showed the same pattern. As ILE levels decreased, both LEU and VAL levels increased. This was an unexpected finding, as human CKD has been previously associated with reduced serum levels of branched-chain amino acid, as the consequence of multiple factors such as metabolic acidosis and low protein diet [18]. This difference may not be attributed exclusively to the use of a prescription renal diet. A previous study showed that dogs fed with prescription renal diet had significantly higher serum levels of LEU and similar levels of VAL, compared to clinically healthy dogs fed with the same diet. This study concluded that CKD dogs at advanced IRIS stage (3–4) showed a significant difference in amino acid metabolism, compared to healthy dogs [5]. Therefore, it is possible that conditions other than dietary composition were responsible for the elevation in serum LEU, LYS, and VAL. A possible explanation may be a condition of insulin resistance. Although the exact mechanism by which insulin resistance promotes the increase in serum branched-chain amino acids is not completely understood, it may interfere with the activity of the branched-chain keto-acid dehydrogenase [18,19]. Additionally, the reduction in serum ALA may be secondary to insulin resistance, as this pathological condition has been reported to be associated with decreased serum levels of gluconeogenic amino acids in humans [18].

Among NEAAs, ALA, CYS, and SER decreased significantly in CKD, while ARG, GLU, GLY, and PRO showed an opposite pattern. The finding of lower serum levels of SER and higher levels of GLY was consistent with previous evidence in human CKD patients [20]. As kidneys are highly responsible for the conversion of GLY to SER, it is plausible that serum GLY levels tend to elevate in CKD. This finding was already reported by a previous study in dogs, in which SER/GLY ratio was directly related to reduced creatinine clearance [21]. The process of conversion of glycine to serine involves glycine cleavage enzyme and serine hydroxy-methyl-transferase in the proximal tubule. A second way of serine production involves the conversion of gluconeogenic precursors, such as glutamine, glutamate, and aspartate to phosphoserine, and then to serine [20]. Therefore, it is possible that the elevated levels of serum GLU of our patients were derived from a partial failure of this mechanism. Another possible reason for GLU increase in our population may be a reduced capacity of GLU uptake from peripheral muscles. In human CKD patients, elevated serum GLU levels have been shown to correlate with reduction in body cell mass [22]. High serum GLU levels have been shown to decrease in human patients following hemodialysis treatment, and GLU accumulation may contribute to uremic encephalopathy [22]. In our cohort of dogs, serum ARG levels were significantly higher compared to healthy dogs, particularly at IRIS stages 3 and 4. ARG levels may increase in CKD due to different reasons. As more than 80% of ARG derives from protein breakdown [23,24], it is possible that dogs experiencing a higher degree of muscle waste showed increased serum levels of ARG. In addition to serum ARG, our CKD dogs also showed elevated serum levels of PRO. A possible explanation for the elevation in PRO may be due to its role as a ROS scavenger. PRO is known to contribute significantly to the homeostasis of the REDOX status during chronic inflammatory conditions [25]. In human CKD patients, elevated serum PRO can also derive from increased muscular release during cachexia [25]. This finding was not in agreement with our results, in which dogs affected by PEW showed significantly lower serum PRO, compared to the nPEW group. One hypothesis for this difference may be related to the fact that PRO is a NEAA, which derives from both endogenous production and dietary sources. Dogs with PEW were unable to cover their daily energy requirements from renal diet. Therefore, we could not exclude that their dietary intake of PRO was reduced. Serum CYS was significantly lower in CKD dogs compared to healthy dogs. CYS showed a pattern similar to human CKD. In those patients, serum CYS appeared significantly lower than healthy subjects, and its reduction seemed to worsen with the progression of the CKD stage. As is known, CYS contributes to maintain oxidant–antioxidant equilibrium, and in CKD humans CYS is supposed to be a quantitative oxidative-stress biomarker [26]. Therefore, it is possible that its serum reduction in CKD dogs was a marker of elevated oxidative stress.

The serum amino-acidic pattern of our dogs did not seem to be significantly affected by the presence of abnormal serum CaxP, with the exception of TRP and CYS. However, it is to be noticed that a non-statistically significant reduction in the median distribution of both EAAs and NEAAs was found. As known, in CKD dogs an abnormal CaxP has been associated with a significant reduction in survival rate [8,27]. As bone and mineral disorders have a significant role in the progression of CKD, the concomitant reduction in serum TRP, and elevation in CYS may be secondary to a worsening of the renal function in dogs with abnormal CaxP. Serum TRP may decrease due to elevation of its catabolites, the most famous of all is indoxyl sulfate [17]. On the other side, the serum elevation of CYS in this group of dogs may be caused by disorders in CYS and homocysteine (HCY) metabolism. Reductions in residual renal function, and particularly in glomerular filtration, have been associated with increased serum levels of both CYS and HCY [28].

In our cohort, metabolic acidosis was associated with a significant reduction in the median distribution of EAAs. This was an expected finding, as metabolic acidosis is a very common cause of lean body mass wasting in human CKD. Metabolic acidosis is the result of both a reduction in the net tubular secretion of protons, and an impaired glomerular elimination of organic acid residues. The loss of lean body mass is mainly mediated by the overstimulation of the intramuscular ubiquitin–proteasome system [29,30]. CKD dogs with metabolic acidosis showed significantly higher serum levels of both PHE and LEU. A possible reason for PHE elevation in this group of dogs may be its mobilization from muscular tissue, due to increased protein breakdown. In physiological conditions, PHE is actively converted to TYR by hydroxylation. In CKD patients PHE levels in both serum and muscle tend to accumulate, due to the reduced activity of the renal phenylalanine hydroxylase [29]. As previously mentioned, serum levels of branched amino acids (LEU included) have been found to decrease during metabolic acidosis [18]. However, it is possible that the elevation of LEU in dogs with metabolic acidosis was secondary to the effects of insulin resistance. Metabolic acidosis has been shown to be a promoting condition for insulin resistance in CKD humans. Correction of metabolic acidosis, by administration of sodium bicarbonate, has been associated with significant improvement in insulin resistance [31]. Therefore, it is plausible that CKD dogs with metabolic acidosis experienced a certain degree of insulin resistance, and serum elevation of LEU.

In our dogs, the presence of PEW affected significantly the median distribution of both EAAs and NEAAs. Their median serum concentrations were approximately half the median concentrations of the non-PEW group. This finding seemed to confirm reports in human medicine, in which PEW is considered one of the most important causes of amino acid deficiency. As known, PEW is defined as a pathological condition, characterized by abnormally low levels or excessive losses of body protein mass and energy reserves. Although PEW may be present at any stage of CKD, its frequency increases with the progression of the disease, with approximately 75% of patients affected at CKD stage 5 [32]. This finding seemed to be similar to our results, in which the frequency of PEW increased, particularly at stage IRIS 4. Although exact incidence and pathophysiology of PEW in CKD dogs are unknown, it is plausible that it has the same causes occurring in human CKD. Therefore, it is possible that the reduction in EAAs and NEAAs in dogs affected by PEW was caused by multiple factors. The abnormal amino-acidic pattern of these dogs might be secondary to inflammatory and hormonal triggers of proteolysis, such as increased inflammatory cytokines and insulin resistance. In addition to these causes, poor body conditions and anorexia might contribute to reduce nutrient intake, thus altering the normal balance between EAAs and NEAAs.

The present study has several limitations. As the study was conducted on leftover serum samples of CKD dogs, data regarding proteinuria and blood pressure measurements were not present for all the dogs. For this reason, we were unable to include these data in the statistical analysis. As there were a relatively low number of dogs included in each IRIS stage, it was not possible to conduct a statistical comparison of different EAAs and NEAAs according to CaxP, metabolic acidosis, and PEW. Dogs of the control group were younger than dogs of the CKD group. Although CKD may also affect young animals, its prevalence increases in aged subjects. Therefore, it is possible that the difference in the mean age affected the distribution of EAAs and NEAAs in the two groups.

Another possible limitation was the non-homogeneity of the diet in both the control and CKD groups. Dogs of the control group were clinically healthy blood donors, which were mostly on commercial maintenance dry or wet food. However, some of the owners occasionally fed them home-cooked food. Although the majority of CKD dogs were fed a prescription renal diet, not all patients were able to consume a 100% renal diet. Especially when poor appetite was present, some of the owners mixed the renal diet with non-renal diet. On the other hand, some of the CKD dogs had an esophagostomy feeding tube, so owners could feed the dogs a 100% renal diet. Therefore, a standardized diet was not possible in our cohort of patients.

## 5. Conclusions

The serum pattern of both EAAs and NEAAs was significantly different in CKD dogs compared to healthy dogs, although no association with the progression of the IRIS stage was found. Abnormal serum concentrations of amino acids in CKD dogs suggested a potential key role of pathological conditions, such as inflammation, insulin resistance, and increased protein break down.

Among different complications of CKD, PEW seemed to affect more severely the serum amino-acidic pattern by causing significant reduction in both EAAs and NEAAs.

## Figures and Tables

**Table 1:** Descriptive statistics of serum creatinine, urea, total calcium, phosphate, CaxP (mg^2^/dL^2^), total protein, albumin, bicarbonate, and pH, expressed as mean ± standard deviation (SD).

	IRIS 1 (*n* = 12)	IRIS 2 (*n* = 16)	IRIS 3 (*n* = 14)	IRIS 4 (*n* = 20)
**Creatinine** (mg/dL)	1.4 ± 0.2	2.0 ± 0.3	2.9 ± 0.8	5.5 ± 2.2
**Urea** (mg/dL)	63.2 ± 22.1	94.9 ± 52.7	184.3 ± 43.2	203.3 ± 80.4
**Calcium** (mg/dL)	9.5 ± 3.4	9.7 ± 1.2	10.1 ± 1.5	11.5 ± 1.16
**Phosphate** (mg/dL)	4.5 ± 1.1	4.8 ± 0.9	5.1 ± 1.2	9.2 ± 4.0
**CaxP** (mg^2^/dL^2^)	46.7 ± 5.6	48.4 ± 0.2	50.7 ± 11.6	103.1 ± 40.8
**Total protein** (g/dL)	6.5 ± 0.7	6.4 ± 0.8	6.0 ± 0.9	6.3 ± 1.0
**Albumin** (g/dL)	3.4 ± 0.4	2.9 ± 0.6	3.1 ± 0.5	2.9 ± 0.4
**Bicarbonate** (mmol/L)	21.0 ± 4.6	21.2 ± 4.9	22.7 ± 5.0	19.1 ± 3.8
**pH**	7.3 ± 0.0	7.3 ± 0.0	7.2 ± 0.0	7.2 ± 0.0

**Table 2:** One-way ANOVA and Kruskal–Wallis comparisons of serum amino acids of dogs of the control group and dogs at different stages of chronic kidney disease.

	CG (*n* = 25)	IRIS 1 (*n* = 12)	IRIS 2 (*n* = 16)	IRIS 3 (*n* = 14)	IRIS 4 (*n* = 20)	*p*-Value
**EAAs**						
**HIS** (nmol/mL)	205.2 ** ± 47.4	113.8 * ± 53.2	133.1 ± 80.0	158.9 ± 68.3	121.4 * ± 83.8	***<0.001***
**PHE** (nmol/mL)	12.6 ± 5.1	11.5 ± 7.6	13.2 ± 7.2	14.1 ± 5.9	13.63 ± 7.7	0.868
**THR** (nmol/mL)	57.6 ± 20.1	36.2 ± 21.5	50.0 ± 19.3	65.1 ± 28.7	48.4 ± 32.2	0.05
**ILE** (nmol/mL)	24.2 ** (12.6–45.4)	7.8 * (3.2–24.4)	8.5 * (3.8–38.6)	11.3 * (2.9–18.2)	7.18 * (1.3–21.1)	***<0.001***
**LEU** (nmol/mL)	10.6 ** (7.7–34.1)	20.5 (6.4–64.8)	20.9 * (9.1–43.2)	25.8 * (5.9–42.1)	18.10 (2.7–34.0	***<0.001***
**LYS** (nmol/mL)	7.2 ** (3.7–64.0)	29.7 * (6.6–97.2)	37.9 * (16.2–102.2)	59.8 * (13.5–99.7)	31.7 * (6.3–94.0)	***<0.001***
**MET** (nmol/mL)	4.7 (0.3–58.4)	4.3 (0.9–11.2)	7.6 (0.2–29.8)	6.6 (2.8–19.5)	4.62 (1.1–19.7)	0.333
**TRP** (nmol/mL)	48.2 ** (19.6–108.3)	16.4 * (3.3–38.0)	16.8 * (7.4–25.0)	15.6 * (6.2–21.3)	13.0 * (1.9–28.1)	***<0.001***
**VAL** (nmol/mL)	4.8 ** (2.9–14.2)	23.6 * (8.8–68.5)	31.0 * (14.0–61.8)	31.3 * (4.1–60.1)	20.9 * (1.9–40.7)	***<0.001***
**ARG** (nmol/mL)	59.2 (11.4–172.5)	42.9 ** (11.8–73.1)	75.5 (25.2–127.8)	100.2 * (31.2–377.0)	88.0 * (17.6–348.0)	***0.002***
**NEAAs**						
**PRO** (nmol/mL)	11.8 ** ± 13.6	37.1 * ± 15.6	38.3 * ± 18.0	47.3 * ± 15.1	32.8 * ± 18.0	***<0.001***
**SER** (nmol/mL)	41.3 ± 14.7	25.5 ± 18.0	29.3 ± 15.1	40.7 ± 17.0	29.9 ± 20.0	***0.002***
**ALA** (nmol/mL)	94.4 ** (56.5–225.9)	68.0 (21.2–180.7)	60.2 (38.6–211.2)	112.1 (20.3–189.8)	61.4 * (6.9–155.3)	***0.013***
**ASP** (nmol/mL)	1.2 (0.1–5.4)	1.4 (0.2–5.1)	1.3 (0.0–7.4)	3.3 (0.4–7.0)	2.39 (0.2–5.3)	0.146
**CYS** (nmol/mL)	38.9 *** (7.6–73.8)	3.5 ** (2.1–4.7)	4.1 * (0.1–8.6)	4.53 * (3.5–8.1)	6.52 ** (0.3–17.6)	***<0.001***
**GLU** (nmol/mL)	8.7 ** (4.3–20.5)	15.9 (6.8–71.4)	18.5 * (4.4–50.3)	22.5 * (3.3–46.2)	14.9 (2.1–37.5)	***0.002***
**GLY** (nmol/mL)	48.8 * (28.2–84.3)	49.1 * (17.3–106.8)	54.5 (21.3–109.4	95.6 ** (29.2–137.5)	55.8 (7.1–130.4)	***0.010***
**TYR** (nmol/mL)	11.3 (6.2–53.9)	7.8 (3.4–15.7)	8.9 (3.6–27.7)	13.0 (4.0–37.9)	9.4 (0.1–36.7)	0.120
**EAA sum** (nmol/mL)	11,158.7	3822.9	6534.9	7085.6	7855.6	
**median** (min-max)	19.5 (0.3–323.4)	20.7 (0.0–185.8)	25.2 (0.0–364.1)	25.72 (2.89–372.8)	17.84 (1.1–348.2)	***0.020***
**NEAA sum** (nmol/mL)	6910.5	2756.5	3940.7	4489.6	4572.4	
**median** (min-max)	24.7 (0.1–225.9	15.8 (0.2–180.7)	21.7 (0.0–211.2)	28.0 (0.4–189.8)	16.3 (0.1–155.3)	0.106

EAAs (essential amino acids): L-Histidine (HIS), L-Isoleucine (ILE), L-Leucine (LEU), L-Lysine (LYS), Methionine (MET), L-Phenylalanine (PHE), L-Threonine (THR), Tryptophan (TRP), L-Valine (VAL), L-Arginine (ARG). Among non-essential amino acids (NEAAs), the following were analyzed: L-Alanine (ALA), L-Aspartic acid (ASP), L-Cysteine (CYS), L-Glutamic acid (GLU), Glycine (GLY), Proline (PRO), L-Serine (SER), L-Tyrosine (TYR); EAA sum (sum of EAAs), NEAA sum (sum of NEAAs), CG: control group. Statistical significance at Bonferroni post-test was indicated with *, ** and ***. CG and CKD dogs differed significantly in terms of HIS, ILE, LEU, LYS, TRP, VAL, and ARG (EAAs), and in terms of PRO, SER, ALA, CYS, GLU, and GLY (NEAAs).

**Table 3:** *T*-test and Mann–Whitney comparison of serum amino between dogs with serum CaxP > 70 mg^2^/dL^2^ and CaxP ≤ 70 mg^2^/dL^2^; dogs with metabolic acidosis (MA) and dogs without metabolic acidosis (nMA); dogs with PEW (PEW) and dogs without PEW (nPEW).

EAAs									
	CaxP		*p*-Value	Metabolic Acidosis		*p*-Value	PEW		*p*-Value
	>70 mg^2^/dL^2^ (*n* = 22)	≤70 mg^2^/dL^2^ (*n* = 40)		MA (*n* = 30)	nMA (*n* = 32)		PEW (*n* = 11)	nPEW (*n* = 51)	
**HIS** (nmol/mL)	130.8 ± 83.4	132.2 ± 70.2	0.928	163.5 ± 87.6	123.8 ± 68.0	0.077	76.4 ± 56.4	143.9 ± 75.1	***0.006***
**PHE** (nmol/mL)	13.8 ± 7.8	12.8± 6.7	0.592	16.7 ± 7.5	12.3 ± 6.6	***0.035***	9.4 ± 6.6	14.05± 7.0	***0.049***
**THR** (nmol/mL)	54.3 ± 32.7	48.0 ± 24.6	0.398	58.2 ± 26.0	48.1 ± 27.1	0.214	30.3 ± 26.8	54.5 ± 26.1	***0.007***
**ILE** (nmol/mL)	8.4 (1.3–14.3)	8.5 (2.9–38.6)	0.141	11.5 (1.3–38.6)	7.5 (1.6–24.4)	0.068	4.3 (1.3–13.3)	9.4 (2.9–38.6)	***0.001***
**LEU** (nmol/mL)	22.6 (2.7–40.2)	20.9 (5.9–64.8)	0.423	28.6 (2.7–43.2)	20.5 (3.3–64.8)	***0.034***	9.1 (2.7–33.7)	22.2 (5.9–64.8)	***0.001***
**LYS** (nmol/mL)	51.2 (6.0–95.0)	37.9 (6.6–102.2)	0.791	54.7 (6.0–99.0)	34.4 (6.6–102.2)	0.159	29.3 (6.0–83.1)	46.8 (6.6–102.2)	***0.014***
**MET** (nmol/mL)	6.6 (1.1–19.7)	6.0 (0.1–29.8)	0.724	7.3 (0.0–17.6)	5.11 (0.0–29.8)	0.682	4.1 (1.1–19.5)	6.8 (0.0–29.8)	0.064
**TRP** (nmol/mL)	13.0 (1.9–28.0)	16.7 (3.3–30.1)	***0.025***	14.7 (1.9–27.2)	16.3 (3.3–30.0)	0.657	7.9 (1.9–19.7)	16.3 (3.3–30.0)	***0.003***
**VAL** (nmol/mL)	25.1 (1.9–60.1)	24.6 (1.7–68.5)	0.384	31.1 (1.9–51.7)	23.3 (4.1–68.5)	0.116	12.8 (1.9–39.8)	27.2 (4.1–68.5)	***0.003***
**ARG** (nmol/mL)	88.0 (17.6–348.2)	72.3 (11.8–372.8)	0.317	100.8 (21.0–233.8)	72.4 (11.8–372.8)	0.085	71.8 (17.6–87.4)	77.6 (11.8–372.8)	0.057
**NEAAs**									
	**CaxP > 70**	**CaxP ≤ 70**	***p*-Value**	**MA**	**nMA**	***p*-Value**	**PEW** **(*n* = 11)**	**nPEW** **(*n* = 51)**	***p*-Value**
**PRO** (nmol/mL)	37.5 ± 17.0	38.7 ± 17.8	0.795	43.7 ± 19.4	36.8 ± 16.7	0.193	26.3 ± 14.2	40.9 ± 17.0	***0.011***
**SER** (nmol/mL)	33.1 ± 19.9	30.4 ± 17.3	0.58	38.6 ± 18.2	29.2 ± 17.4	0.078	20.7 ± 16.1	33.6 ± 17.9	***0.031***
**ALA** (nmol/mL)	65.4 (6.9–155.3)	67.5 (20.3–211.2)	0.206	100.1 (8.01–183.7)	63.3 (6.9–211.2)	0.296	34.9 (6.9–136.4)	77.6 (11.8–372.8)	***0.001***
**ASP** (nmol/mL)	2.4 (0.2–7.0)	1.8 (0.1–7.4)	0.883	3.0 (0.2–7.0)	1.7 (0.0–7.4)	0.057	0.8 (0.2–4.8)	2.4 (0.0–7.4)	0.074
**CYS** (nmol/mL)	6.0 (0.3–17.6)	4.1 (0.1–9.0)	***0.003***	4.9 (3.4–17.6)	4.6 (0.1–16.0)	0.375	5.7 (4.3–15.9)	4.2 (0.1–17.6)	***0.010***
**GLU** (nmol/mL)	16.8 (2.1–71.4)	18.0 (3.3–50.3)	0.680	18.3 (2.1–71.4)	16.9 (3.0–50.3)	0.375	8.7 (2.1–46.0)	17.7 (3.3–71.4)	***0.002***
**GLY** (nmol/mL)	78.6 (7.1–130.4)	60.2 (17.3–137.0)	0.270	83.4 (9.9–129.3)	60.2 (7.1–137.0)	0.139	45.5 (7.1–102.5)	63.6 (17.3–137.0)	***0.045***
**TYR** (nmol/mL)	10.4 (0.1–36.7)	9.35 (3.49–37.90)	0.883	10.8 (0.1–27.7)	9.0 (2.8–37.9)	0.330	5.5 (0.1–18.5)	10.3 (3.4–37.9)	***0.016***
**EAA sum** (nmol/mL)	9048.6	16,139.4		7371.5	17,036.7		2728.3	22,570.7	
**median** (min-max)	18.1 (1.1–348.2)	23.8 (0.1–372.8)	0.146	20.7 (0.0–372.8)	27.2 (0.1–364.1)	***0.034***	12.5 (1.1–170.3)	24.2 (0.0–372.8)	***0.000***
**NEAA sum** (nmol/mL)	5425.4	10,257.2		4426.3	10,751.3		1807.2	13,952.2	
**median** (min-max)	16.4 (0.1–180.7)	20.4 (0.0–211.2)	0.4	18.4 (0.0–211.2)	21.3 (0.09–183.6)	0.1	9.6 (0.1–136.4)	21.0 (0.0–211.2)	***0.001***

Normal: dogs with CaxP < 70 mg^2^/dL^2^; Abnormal: dogs with CaxP ≥ 70 mg^2^/dL^2^. MA: dogs with metabolic acidosis; nMA: dogs without metabolic acidosis; PEW: dogs with PEW; nPEW: dogs without PEW. Dogs with abnormal and dogs with normal CaxP differed significantly in terms of TRP (EAAs) and in terms of CYS (NEAAs); dogs with MA and dogs with nMA differed significantly in terms of PHE and LEU (EAAs), and in terms of EAA sum. Dogs with PEW and dogs with nPEW differed significantly in terms of HIS, PHE, THR, ILE, LEU, LYS, TRP, and VAL (EAAs), and in terms of PRO, SER, ALA, CYS, GLU, GLY, and TYR (NEAAs), and EAA sum and NEAA sum.

## Data Availability

Data generated from the study are available in an electronical database.
